# Synthesis and Application of Fe_3_O_4_–Modified Nano-Perlite as a Dispersive Sorbent for Co(II) Ion Preconcentration

**DOI:** 10.1021/acsomega.6c01728

**Published:** 2026-05-14

**Authors:** Ali Kilicer

**Affiliations:** Department of Geological Engineering, Faculty of Engineering, Van Yüzüncü Yıl University 65080, Van, Turkey

## Abstract

In this study, acid-treated lamellar perlite (ALP) and Fe_3_O_4_-decorated acid-treated lamellar perlite (Fe_3_O_4_-ALP) were synthesized and applied as sorbents for dispersive solid-phase extraction coupled with flame atomic absorption spectrometry (DSPE-FAAS) for trace Co­(II) determination. The synthesized materials were characterized by X-ray diffraction (XRD), Fourier transform infrared spectroscopy (FTIR), scanning electron microscopy coupled with energy-dispersive X-ray spectroscopy (SEM–EDX), and BET surface area analysis. Fe_3_O_4_ decoration onto the lamellar aluminosilicate framework of perlite yielded a mesoporous composite with enhanced surface heterogeneity and convenient magnetic recoverability, facilitating rapid phase separation in the DSPE workflow. Comparative experiments demonstrated that Fe_3_O_4_-ALP exhibited substantially higher extraction efficiency than ALP, confirming the beneficial role of Fe_3_O_4_ decoration on Co­(II) uptake. Under optimized analytical conditions, the DSPE-FAAS method showed a linear response for Co­(II) in the range of 5–250 ng mL^–1^ with a correlation coefficient exceeding 0.999. The limits of detection and quantification were determined as 0.626 ng mL^–1^ and 2.086 ng mL^–1^, respectively, corresponding to a 122-fold LOD improvement factor relative to direct FAAS analysis. The method exhibited satisfactory precision, with relative standard deviations ranging from 4.4% to 8.1%. The applicability of the developed DSPE-FAAS method was validated by analyzing groundwater and rock leachate samples, which yielded recoveries in the range of 84.4–110.4%, confirming method robustness under geochemically complex matrix conditions. Overall, Fe_3_O_4_-ALP represents a cost-effective and analytically efficient sorbent for trace Co­(II) determination in environmental samples.

## Graphical abstract

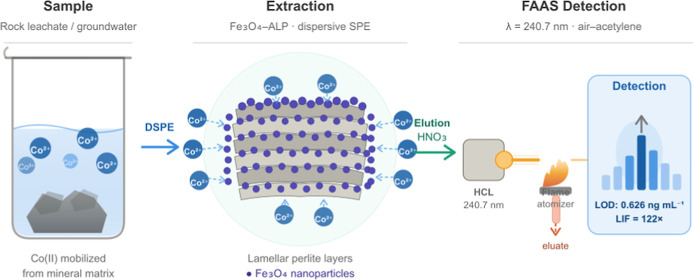

## 1 Introduction

Cobalt (Co) is a strategically important transition metal whose environmental occurrence and behavior are tightly coupled to geological processes. In natural systems, Co is hosted in mafic–ultramafic lithologies, lateritic weathering profiles, and a variety of Co-bearing ore deposits; it can be mobilized to soils and waters through rock–water interaction, weathering, and mining-related disturbances.^1^ Recent geometallurgical and environmental geochemistry literature highlights that Co distributions, speciation, and mobility are strongly controlled by mineralogy (e.g., association with Fe/Mn (oxyhydr)­oxides, sulfides, and secondary phases), redox conditions, and sorption/complexation pathways in mine-affected and natural settings.^2^ These controls make Co a relevant analyte for trace-level determination in geochemically complex natural waters.^3^

From an analytical standpoint, dissolved Co is often present at trace to ultratrace levels in natural waters, particularly in groundwater, spring water, and surface waters that interact with volcanic or ultramafic rocks, or that receive inputs from mine drainage and tailings seepage.^4^ Reliable quantification, therefore, requires methods that can tolerate complex matrices (high ionic strength, competing metals, dissolved organic matter) while delivering low detection limits. In practice, flame atomic absorption spectrometry (FAAS) remains attractive for routine monitoring due to its accessibility and operational simplicity; however, FAAS can be limited by sensitivity when cobalt concentrations approach the ng mL^–1^ range, motivating an upstream preconcentration/separation step before instrumental determination.^5^

Among sample preparation strategies, dispersive solid-phase extraction (DSPE) has gained increasing attention because it minimizes solvent use, reduces processing time, and enables rapid phase separation—particularly when magnetically recoverable sorbents are employed to facilitate fast, filtration-free analyte collection.^6^ In DSPE, the sorbent is dispersed directly into the sample to maximize contact area and adsorption kinetics, and the magnetically recoverable sorbent is then separated using an external magnet or centrifugation. This approach has been increasingly adopted for trace metal analysis in food and water matrices, where fast handling and matrix cleanup are critical.^7^ These features are particularly relevant for Co determination, where the analyte can coexist with high levels of Fe, Mn, Ni, Cu, Zn, Ca, Mg, and organic ligands depending on the geological setting. Consequently, developing efficient DSPE sorbents that combine (i) strong Co­(II) affinity, (ii) rapid adsorption kinetics, and (iii) convenient phase separation remains an active and practically important area.

Natural aluminosilicate minerals offer a compelling platform for magnetic sorbent design because they are abundant, low-cost, and geologically meaningful—allowing the analytical workflow to be framed within an environmental geochemistry context. Perlite, a hydrated volcanic glass with predominantly siliceous composition and exchangeable cations, has long been used as an industrial mineral and has been increasingly studied as an adsorbent for pollutants.^8^ Its adsorption behavior is commonly attributed to surface hydroxyl groups, aluminosilicate framework properties, and modifiable surface charge. A key advantage for trace-metal preconcentration is that perlite can be chemically and thermally activated to increase accessible adsorption sites, enhancing its sorption performance for target metal ions.^8b,9^

However, pristine perlite is not magnetically recoverable, and conventional perlite-based adsorbents are typically applied in batch adsorption or fixed-bed formats rather than in rapid DSPE workflows designed for trace analysis. To bridge this limitation, Fe_3_O_4_ coating has emerged as an effective strategy to introduce additional surface functionality associated with iron-oxide domains known to strongly bind metal ions. Indeed, Fe_3_O_4_-modified perlite has been reported to improve removal performance for certain inorganic contaminants (e.g., Cr­(VI)) compared with unmodified perlite, supporting the premise that iron-oxide functionalization can significantly enhance adsorption efficiency.^10^ Yet, despite these advances, the application of acid-activated lamellar nanoperlite as a sorbent in a DSPE-FAAS workflow for trace Co­(II) determination has not been reported in the literature. Existing studies on perlite-based sorbents for trace metals have focused on batch adsorption capacity or conventional SPE formats; none have evaluated a nanostructured, Fe_3_O_4_-decorated acid-treated perlite composite in a DSPE-FAAS procedure for Co­(II) in geochemically relevant matrices such as groundwater and rock leachates. The three principal novelties of the present work are (i) the lamellar nanoperlite architecture produced by acid activation and calcination as an analytically optimized sorbent platform, (ii) the DSPE-FAAS workflow enabling rapid, filtration-free preconcentration with a 122-fold LOD improvement factor, and (iii) validation in groundwater and rock leachate matrices.

In this study, we report the synthesis of acid-treated lamellar nanoperlite (ALP) and its Fe_3_O_4_-decorated analogue (Fe_3_O_4_-ALP) and their application as sorbents in a DSPE-FAAS procedure for trace Co­(II) determination. The nanoperlite precursor was produced through acid-assisted treatment under elevated temperature followed by calcination, yielding a lamellar aluminosilicate framework with enhanced surface accessibility. Fe_3_O_4_ deposition was subsequently employed to introduce iron-oxide surface functionality and to enable convenient magnetic phase separation within the DSPE workflow. The extraction performance of Fe_3_O_4_-ALP was systematically compared with that of ALP to elucidate the contribution of Fe_3_O_4_ decoration to Co­(II) preconcentration efficiency. The materials were characterized by SEM–EDX, FTIR, BET surface area analysis, and XRD to relate textural and surface properties to adsorption behavior. The proposed DSPE-FAAS method was optimized and validated in terms of linearity, sensitivity, precision, and LOD improvement factor, and its applicability was demonstrated using groundwater and rock leachate samples.

## 2 Experimental Section

### 2.1 Materials

Natural perlite was supplied from a volcanic deposit in Türkiye, and the raw perlite was used as the starting geological material and subjected to chemical and thermal treatments to obtain acid-activated nanoperlite. All reagents used in this study were of analytical grade and were employed without further purification. Iron­(III) chloride hexahydrate (FeCl_3_·6H_2_O), iron­(II) chloride tetrahydrate (FeCl_2_·4H_2_O), hydrochloric acid (HCl), nitric acid (HNO_3_), and ammonia solution (NH_3_, 25%) were obtained from Merck (Darmstadt, Germany). Deionized water (18.2 MΩ cm) was used throughout all experiments.

A stock cobalt­(II) solution (1000 mg L^–1^) was obtained as a certified ICP standard solution from High-Purity Standards (USA). Working standard solutions were freshly prepared daily by appropriate dilution of the stock solution. Buffer solutions were prepared using potassium hydrogen phthalate (pH 3–6), tris­(hydroxymethyl)­aminomethane (Tris, pH 7), borax (pH 8–10), and disodium hydrogen phosphate (pH 11–12) (Merck, Darmstadt, Germany). The pH of the solutions was adjusted as required using dilute HCl and NaOH.

### 2.2 Instrumentation

Ultrapure water obtained from a Merck Millipore Direct-Q 3 UV purification system was used for the preparation of calibration and working standard solutions. Cobalt determinations were performed using a flame atomic absorption spectrophotometer (Thermo Scientific ICE-3000 series, USA) equipped with a deuterium background correction system. A cobalt hollow cathode lamp (HCL) was used as the radiation source, and absorbance measurements were carried out at an analytical wavelength of 240.7 nm under the manufacturer’s recommended operating conditions. An air–acetylene flame was employed for all FAAS measurements.

### 2.3 Preparation of Acid-Treated Lamellar Perlite

Raw perlite was initially washed five times with deionized water to remove loosely bound impurities and then dried at 100 °C for 8 h. A portion of the dried material (5.0 g) was subjected to thermal pretreatment at 500 °C for 1 h to eliminate volatile components and to promote partial dehydroxylation of the aluminosilicate framework. The thermally treated perlite was subsequently refluxed in 2.0 mol L^–1^ HCl solution for 24 h under continuous stirring.^9,11^ This acid activation step was employed to leach exchangeable and weakly bound cations and to induce surface restructuring, increasing the density of accessible surface sites on the aluminosilicate framework.

After acid treatment, the suspension was filtered, and the solid residue was repeatedly washed with deionized water until a neutral pH was attained, ensuring complete removal of residual acid. The acid-treated material was dried at 100 °C for 1 h and further calcined at 700 °C for 2 h to obtain a thermally stabilized, acid-treated lamellar perlite (ALP). The resulting material exhibited a plate-like lamellar morphology characteristic of acid-activated aluminosilicate minerals. Morphological features and particle-scale characteristics were examined by scanning electron microscopy (SEM).

### 2.4 Synthesis of Fe_3_O_4_–Decorated Acid-Treated Lamellar Perlite (Fe_3_O_4_-ALP)

Magnetic functionalization of ALP was carried out via a coprecipitation approach to deposit magnetite (Fe_3_O_4_) onto the perlite surface. Briefly, FeCl_3_·6H_2_O (0.05 mol L^–1^) and FeCl_2_·4H_2_O (0.025 mol L^–1^), maintaining a molar ratio of Fe^3+^ to Fe^2+^ of 2:1, were dissolved in deionized water. Acid-treated lamellar perlite (ALP) was added to the iron salt solution at a concentration of 5.0 g L^–1^, and the suspension was heated to 80 °C under continuous stirring.

Magnetite formation was induced by the dropwise addition of 1.0 mol L^–1^ NaOH solution under a nitrogen atmosphere until the pH reached 8.0–8.5. The coprecipitation process resulted in the formation of Fe_3_O_4_ nanoparticles, which were simultaneously immobilized onto the ALP surface. The resulting brown magnetic composite was separated by centrifugation, washed several times with deionized water to remove unreacted species, and dried under ambient conditions.^12^

The successful deposition of magnetite was preliminarily indicated by a distinct color change of the material from off-white (ALP) to dark brown (Fe_3_O_4_-ALP), and subsequently confirmed by comprehensive characterization using SEM–EDX, FTIR, BET surface area analysis, and XRD.

### 2.5 Characterizations

The structural and phase properties of Fe_3_O_4_, ALP, and Fe_3_O_4_-ALP were investigated by X-ray diffraction (XRD) using Cu Kα radiation to identify crystalline phases and to confirm the formation of magnetite in the composite material. The morphology and surface features of the synthesized sorbents were examined by scanning electron microscopy (SEM). For the magnetically modified material, the elemental composition and iron-oxide distribution were further evaluated by energy-dispersive X-ray spectroscopy (EDX). Fourier transform infrared spectroscopy (FTIR) was employed to assess changes in surface functional groups associated with acid treatment and magnetic decoration. The textural properties, including specific surface area and pore characteristics, were determined for both ALP and Fe_3_O_4_-ALP materials by nitrogen adsorption–desorption measurements using the Brunauer–Emmett–Teller (BET) method. Together, these techniques provided complementary information on the structural, morphological, elemental, and surface properties relevant to the extraction performance of the synthesized materials.

### 2.6 Dispersive Solid-Phase Extraction (DSPE) Procedure

The DSPE-FAAS procedure developed for Co­(II) determination was applied for optimization studies, analytical performance evaluation, and recovery experiments in real samples. Standard Co­(II) solutions were prepared by appropriate dilution of a 1000 mg L^–1^ stock solution.

For the extraction step, a defined volume of standard or real sample solution was adjusted to the optimum pH using a suitable buffer. A predetermined amount of ALP or Fe_3_O_4_-ALP sorbent was then dispersed into the solution and mixed for the optimized time to promote effective interaction between the sorbent surface and Co­(II) ions.

Following extraction, the suspension was centrifuged to promote sedimentation of the sorbent particles and facilitate phase separation. After decantation of the supernatant, an external magnet was applied to the collected particles to maintain stable retention of the solid phase during the subsequent decantation and handling steps.

Desorption of Co­(II) was achieved by adding an appropriate volume of acidic eluent to the collected particles and mixing for the optimized time. After the desorption step, centrifugation and an external magnet were reapplied to retain the solid phase and facilitate clean separation of the analyte-containing eluent before FAAS measurement under optimized instrumental conditions.

Key parameters affecting extraction efficiency, including solution pH, sorbent type and amount, eluent type and volume, and mixing conditions, were systematically optimized. The analytical performance of the method was evaluated in terms of linearity, sensitivity, and precision using standard solutions and replicate measurements.

### 2.7 Preparation of the Real Samples

Groundwater and rock leachate samples were used to assess the applicability of the proposed DSPE-FAAS method under geochemically relevant conditions. Groundwater samples were collected from different locations (Tusba, Edremit, and Ipekyolu) in Van using clean polyethylene bottles. Upon arrival at the laboratory, the samples were filtered through a 0.45 μm membrane filter to remove suspended particulate matter. To minimize potential matrix effects arising from high ionic strength and dissolved constituents, the groundwater samples were diluted 50-fold with deionized water before analysis. The diluted samples were stored at 4 °C and analyzed within a short period.

Rock leachate samples were prepared to simulate cobalt release from geological materials through water–rock interaction processes. Representative rock samples were first crushed and ground to obtain a homogeneous powder. An accurately weighed amount of the powdered rock (5.0 g) was transferred into a polyethylene vessel, and 100 mL of deionized water was added, corresponding to a solid-to-liquid ratio of 1:20 (w/v). The suspension was agitated on a mechanical shaker at ambient temperature for 24 h to promote leaching of cobalt from the solid matrix. After equilibration, the suspension was filtered to separate the solid residue, and the resulting leachate was collected for further analysis.

Before the extraction procedure, the pH of all real samples was adjusted to the optimized value using an appropriate buffer system. The DSPE-FAAS method was then applied following the optimized protocol. Recovery studies were carried out by spiking the groundwater and rock leachate samples with known concentrations of Co­(II) to evaluate matrix effects and method accuracy.

## 3 Results and Discussion

### 3.1 Characterization of ALP and Fe_3_O_4_-ALP

The physicochemical properties of acid-treated lamellar perlite (ALP) and Fe_3_O_4_-decorated acid-treated lamellar perlite (Fe_3_O_4_-ALP) were examined to clarify the structural and surface characteristics relevant to their extraction performance toward Co­(II). Emphasis was placed on morphological features and elemental composition, as these factors directly influence surface accessibility and interaction between the sorbent and metal ions during the DSPE process.

Scanning electron microscopy (SEM) images of ALP and Fe_3_O_4_-ALP are shown in Figure 1. The SEM micrographs of ALP (Figure 1a) display a well-defined lamellar morphology composed of plate-like aluminosilicate sheets with relatively smooth surfaces and sharp edges. This layered structure is characteristic of perlite subjected to acid treatment and thermal activation, which promotes partial delamination and exposure of internal surfaces. Such lamellar architectures are advantageous for adsorption-based applications because they provide accessible surface sites and facilitate rapid mass transfer.

**Figure 1:**
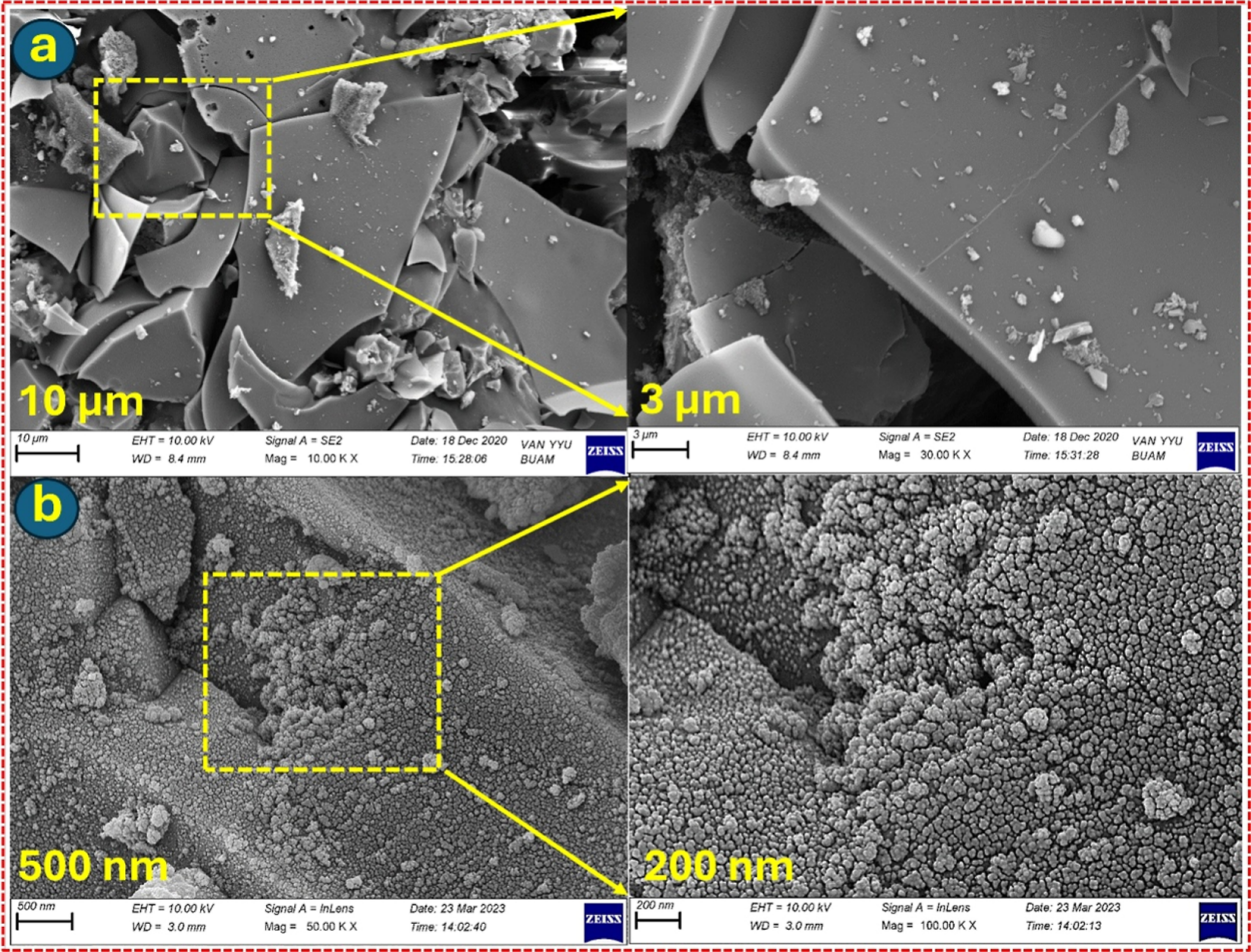
SEM images of (a) acid-treated lamellar perlite (ALP) and (b) Fe_3_O_4_-decorated acid-treated lamellar perlite (Fe_3_O_4_-ALP).

Following surface modification, the SEM images of Fe_3_O_4_-ALP (Figure 1b) reveal a distinct change in surface texture while preserving the underlying lamellar framework. The perlite sheets are uniformly covered with fine, quasi-spherical nanoparticles, resulting in a noticeably rougher and more granular surface. The homogeneous distribution of these nanoparticles over the perlite surface indicates successful deposition without compromising the structural integrity of the aluminosilicate matrix. This morphological evolution is expected to increase surface heterogeneity and the number of available adsorption sites, which is beneficial for metal-ion uptake.

The elemental composition of Fe_3_O_4_-ALP was further investigated by energy-dispersive X-ray spectroscopy (EDX), and the corresponding spectrum and quantitative data are presented in Figure 2a. The EDX results confirm the presence of oxygen, silicon, and aluminum as the major components, consistent with the aluminosilicate nature of perlite. In addition, a pronounced iron signal is observed, evidencing the incorporation of iron oxide on the perlite surface. Quantitative analysis indicates that iron accounts for approximately 20 wt % of the composite, reflecting effective surface decoration. Minor potassium content is also detected, which is commonly associated with natural perlite minerals.

**Figure 2:**
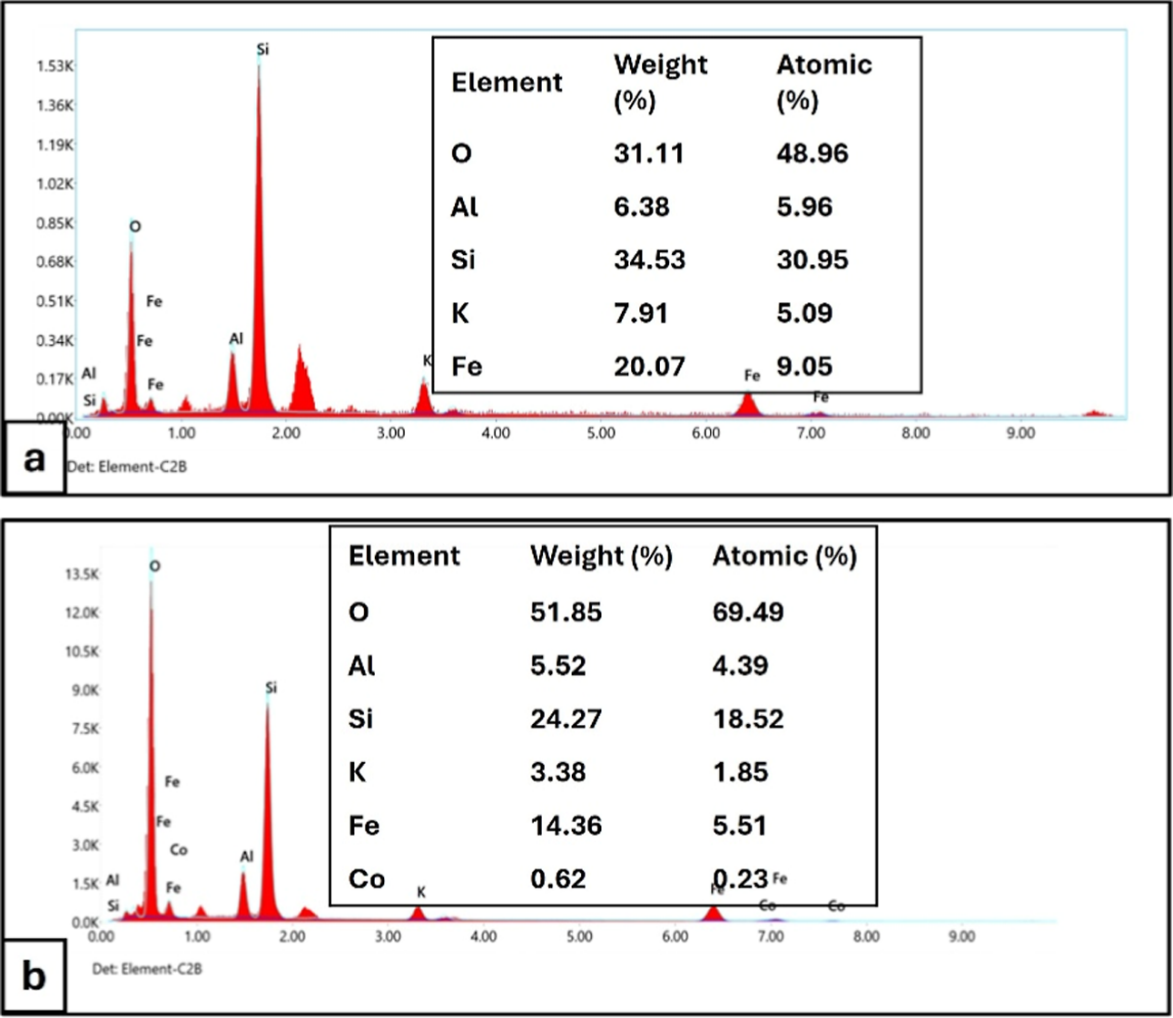
EDX spectra and elemental composition of Fe_3_O_4_-ALP: (a) before Co­(II) adsorption and (b) after Co­(II) adsorption. The appearance of the Co signal in the postadsorption spectrum confirms successful uptake of Co­(II) ions onto the sorbent surface.

To assess the elemental distribution of Co and Fe following the extraction procedure, EDX analysis was additionally performed on Fe_3_O_4_-ALP after Co­(II) adsorption (Figure 2b). The postadsorption EDX spectrum reveals the emergence of a distinct cobalt signal, with Co accounting for 0.62 wt % (0.23 at %) of the sorbent surface composition. This finding provides direct elemental evidence that Co­(II) ions are retained on the sorbent surface following the DSPE procedure. Concurrently, the Fe signal decreased from 20.07 wt % (preadsorption) to 14.36 wt % (postadsorption), which may be attributed to partial surface coverage by adsorbed Co species and associated changes in the probed surface layer. The O, Si, and Al signals, characteristic of the aluminosilicate perlite framework, remain detectable in both spectra, confirming that the structural integrity of the Fe_3_O_4_-ALP composite is maintained throughout the adsorption process. These results collectively support the role of the Fe_3_O_4_-ALP surface as an effective binding site for Co­(II) ions under the optimized DSPE conditions.

FTIR spectroscopy was employed to investigate the surface functional groups of Fe_3_O_4_-ALP, and the spectrum is presented in Figure 3a. The broad absorption band observed in the 3600–3200 cm^–1^ region is attributed to O–H stretching vibrations of surface hydroxyl groups and adsorbed water molecules, which are commonly present on aluminosilicate and iron-oxide surfaces. The band near ∼1040 cm^–1^ corresponds to Si–O–Si asymmetric stretching vibrations, confirming the preservation of the silicate framework after magnetic decoration. Additional bands in the region of 800–600 cm^–1^ are associated with Si–O bending modes, while the absorption bands at ∼543 and ∼694 cm^–1^ are assigned to Fe–O stretching vibrations characteristic of the magnetite phase, providing further evidence for the presence of iron oxide on the composite surface.

**Figure 3:**
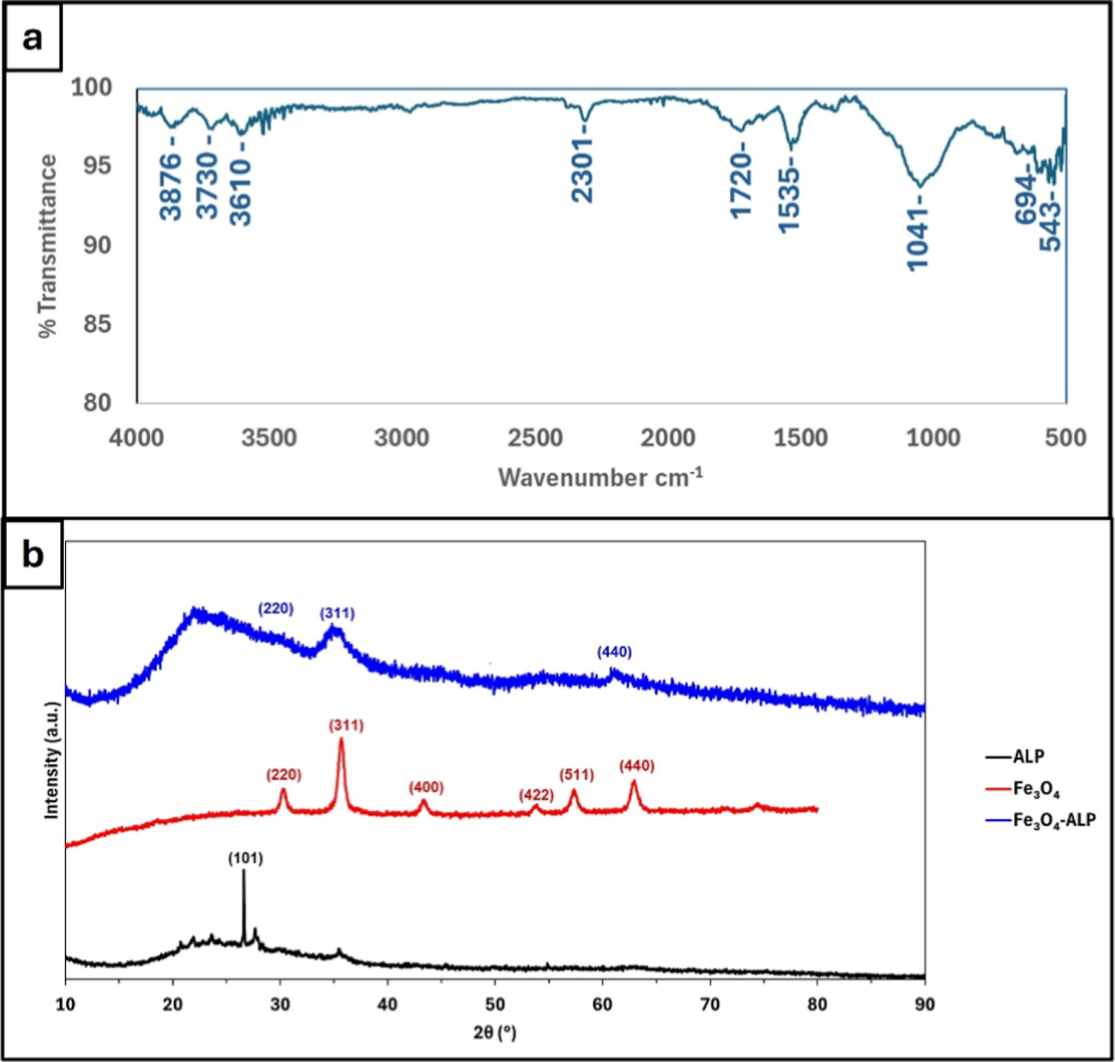
Spectroscopic and structural characterization of Fe_3_O_4_-ALP: (a) FTIR spectrum of Fe_3_O_4_-ALP; (b) XRD patterns of ALP (black), Fe3O4 (red), and Fe3O4-ALP (blue).

The FTIR results indicate that acid treatment and subsequent Fe_3_O_4_ decoration modify the surface chemistry of perlite without destroying its fundamental aluminosilicate structure. The coexistence of silicate-related bands and Fe–O vibrations suggests that both mineral components contribute to the surface properties of Fe_3_O_4_-ALP.

The XRD pattern of acid-treated lamellar perlite (ALP) is presented in Figure 3b alongside those of Fe_3_O_4_ and Fe_3_O_4_-ALP to enable direct comparison of the structural changes induced by Fe_3_O_4_ decoration. The ALP pattern is dominated by a broad, diffuse scattering halo centered between 15° and 35° (2θ), which is characteristic of the amorphous aluminosilicate framework of volcanic glass.^13^ A single sharp reflection observed at ∼26.6° (2θ), indexed to the (101) plane, is attributed to residual crystalline quartz (SiO_2_) present in the natural perlite precursor. The absence of additional sharp reflections confirms that acid treatment and calcination did not induce crystallization of secondary phases within the aluminosilicate matrix.

The crystalline structure and phase composition of Fe_3_O_4_-ALP were further examined by X-ray diffraction (XRD), and the corresponding diffraction patterns are shown in Figure 3b together with that of Fe_3_O_4_ for comparison. The XRD pattern of Fe_3_O_4_ displays sharp and well-defined reflections characteristic of the cubic spinel structure, with prominent peaks indexed to the (220), (311), (400), (422), (511), and (440) planes. These reflections are in good agreement with standard magnetite diffraction data, confirming the crystalline nature of the iron oxide phase.^14^ The presence of the characteristic spinel reflections is consistent with the inverse spinel structure of Fe_3_O_4_, in which Fe^2+^ and Fe^3+^ ions occupy octahedral sites and Fe^3+^ ions occupy tetrahedral sites in a 1:2 ratio.^15^ This crystallographic evidence confirms the coexistence of both oxidation states within the synthesized iron oxide phase, which is relevant to the surface reactivity and metal-binding capacity of Fe_3_O_4_-ALP.

The XRD pattern of Fe_3_O_4_-ALP exhibits several of the characteristic magnetite reflections, most prominently the (220), (311), and (440) planes, indicating partial retention of the Fe_3_O_4_ crystalline structure after deposition onto the acid-treated lamellar perlite surface. The remaining reflections are significantly broadened or attenuated relative to the pure Fe_3_O_4_ pattern, likely reflecting reduced crystallite size, lower Fe_3_O_4_ loading within the composite, or peak overlap with the broad amorphous background of the aluminosilicate support. In addition to the magnetite peaks, a broad diffuse background is observed, consistent with the amorphous aluminosilicate framework of the perlite support.^16^ The coexistence of discernible Fe_3_O_4_ reflections and the amorphous silicate halo is consistent with successful anchoring of magnetite onto the perlite matrix.

Comparison of the three patterns reveals a clear structural progression: the broad amorphous halo of ALP, the sharp spinel reflections of Fe_3_O_4_, and the superposition of both features in Fe_3_O_4_-ALP. The retention of the magnetite reflections in the composite, combined with the persistence of the amorphous perlite background, confirms that Fe_3_O_4_ nanoparticles were successfully anchored onto the ALP surface without inducing crystallization of the aluminosilicate support or formation of secondary iron oxide phases.

The textural properties of both ALP and Fe_3_O_4_-ALP were evaluated by nitrogen adsorption–desorption measurements, and the corresponding isotherms together with the derived parameters are presented in Figure 4 and Table 1. The ALP precursor exhibited a BET surface area of 2.60 m^2^ g^–1^, a total pore volume of 0.01 cm^3^ g^–1^, and a mean pore diameter of 17.07 nm, reflecting its predominantly macroporous character and inherently limited surface accessibility. The low surface area is consistent with the glassy aluminosilicate framework of volcanic perlite, which, despite acid activation and calcination, retains a relatively dense structure with few accessible mesopores.

**Figure 4:**
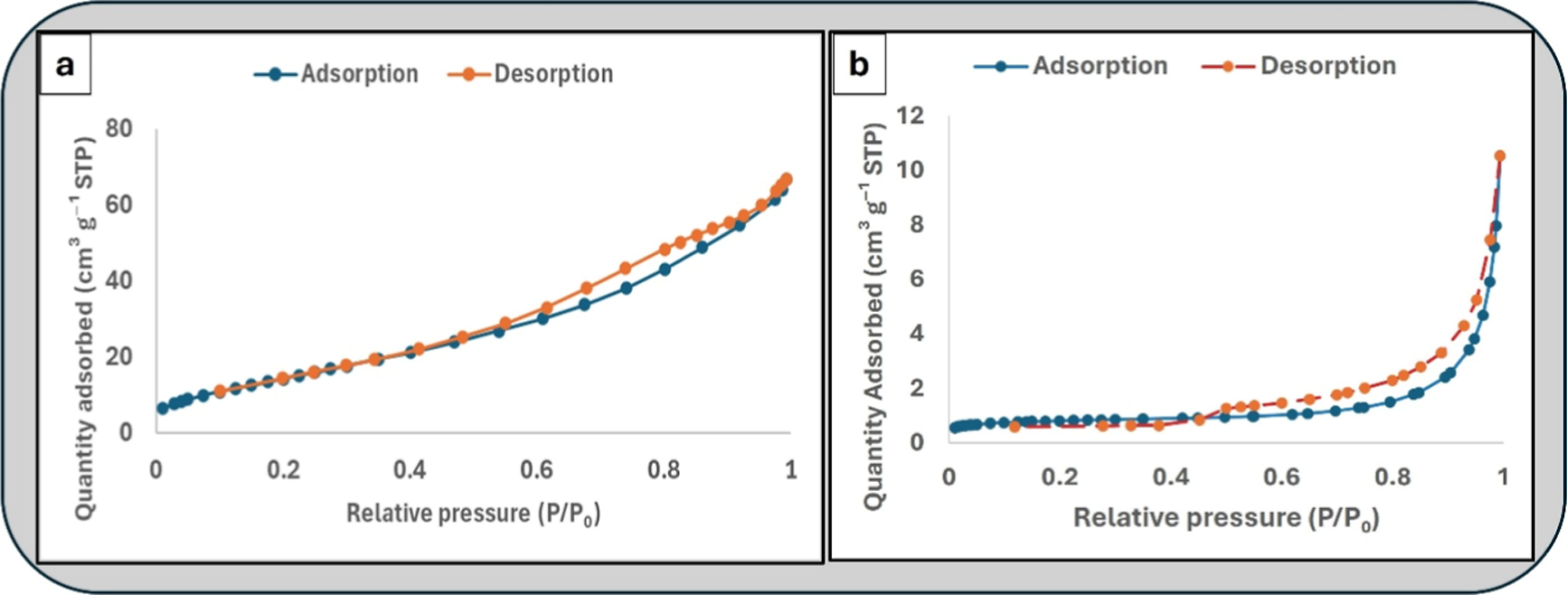
N_2_ adsorption–desorption isotherms of (a) Fe_3_O_4_-ALP and (b) ALP, together with the corresponding textural parameters derived from BET and BJH analyses (Table 1).

**Table 1:** Textural Properties of ALP and Fe_3_O_4_-ALP Determined from N_2_ Adsorption–Desorption Analysis^a^

textural parameter	Fe_3_O_4_-ALP	ALP
*S*_BET_ (m^2^ g^–1^)	47.13	2.60
total pore volume (cm^3^ g^–1^)	0.0903	0.01
average pore diameter (nm)	7.67	17.07
BJH adsorption cumulative pore volume (cm^3^ g^–1^)	0.1034	0.0116

a BET, Brunauer–Emmett–Teller method; BJH, Barrett–Joyner–Halenda method. Average pore diameter calculated by the 4 V/A method from adsorption data.

Following Fe_3_O_4_ decoration, the adsorption–desorption isotherm of Fe_3_O_4_-ALP exhibits a characteristic Type IV profile with a discernible hysteresis loop at intermediate to high relative pressures, indicative of well-developed mesoporosity (Figure 4a). In contrast, the ALP isotherm displays a Type II/III profile with negligible uptake at low to intermediate pressures and a steep rise only near saturation (Figure 4b), consistent with a macroporous, low-surface-area material. Isotherm classification follows the IUPAC recommendations of Thommes et al.^17^

The BET surface area increased approximately 18-fold upon Fe_3_O_4_ decoration, from 2.60 to 47.13 m^2^ g^–1^, accompanied by an 8-fold increase in total pore volume (0.01 to 0.0903 cm^3^ g^–1^) and a shift in dominant pore size from the macroporous range (17.07 nm) into the mesoporous regime (7.67 nm). BJH analysis confirmed a cumulative mesopore volume of 0.1034 cm^3^ g^–1^ for Fe_3_O_4_-ALP, supporting the presence of an interconnected mesopore network generated by the aggregation of iron oxide nanoparticles on the perlite surface. These results collectively demonstrate that Fe_3_O_4_ deposition substantially transforms the textural character of the ALP support, generating a mesoporous composite architecture that is favorable for rapid Co­(II) diffusion and effective surface contact within the DSPE workflow.

### 3.2 Analytical Method Optimization

The analytical performance of the proposed DSPE-FAAS method depends on several experimental variables that influence the adsorption–desorption equilibrium between Co­(II) ions and the sorbent surface. To establish the most favorable operating conditions, a systematic optimization study was carried out using a univariate (one-factor-at-a-time) approach. In this strategy, each parameter was varied individually while all other experimental conditions were kept constant, allowing direct evaluation of its effect on extraction efficiency.

Key parameters affecting the overall extraction performance—including sorbent type (ALP and Fe_3_O_4_-ALP), solution pH, sorbent amount, mixing type and duration, and eluent type and volume—were systematically optimized. The optimization process was directed toward maximizing signal enhancement and improving detection performance following extraction.

#### 3.2.1 Optimization of Sorbent Type

A marked enhancement in analytical signal was observed after the extraction step, confirming the strong dependence of method performance on sorbent type. The comparative experiments were performed using a 40 mL sample volume and 30 mg of each sorbent under natural pH conditions, ensuring consistent evaluation parameters for both materials.

Under these conditions, ALP provided a 26-fold signal enhancement relative to direct measurement, whereas Fe_3_O_4_-ALP achieved a 45-fold enhancement, clearly demonstrating the benefit of Fe_3_O_4_ decoration on Co­(II) extraction efficiency.

The superior signal amplification obtained with Fe_3_O_4_-ALP is attributed to the synergistic integration of iron-oxide domains onto the aluminosilicate framework, which increases the density of surface-active sites and strengthens analyte–sorbent interactions. These results confirm that Fe_3_O_4_ decoration substantially improves Co­(II) extraction efficiency within the DSPE-FAAS workflow and justify the selection of Fe_3_O_4_-ALP for subsequent parameter optimization.

#### 3.2.2 Optimization of pH

The influence of solution pH on Co­(II) extraction efficiency was investigated over the pH range of 2–9, as shown in Figure 5a. The analytical signal increased progressively from strongly acidic conditions toward near-neutral pH values, reaching a maximum at approximately pH 8. A slight plateau behavior was observed between pH 8 and 9, indicating that equilibrium adsorption conditions were attained in this region.

**Figure 5:**
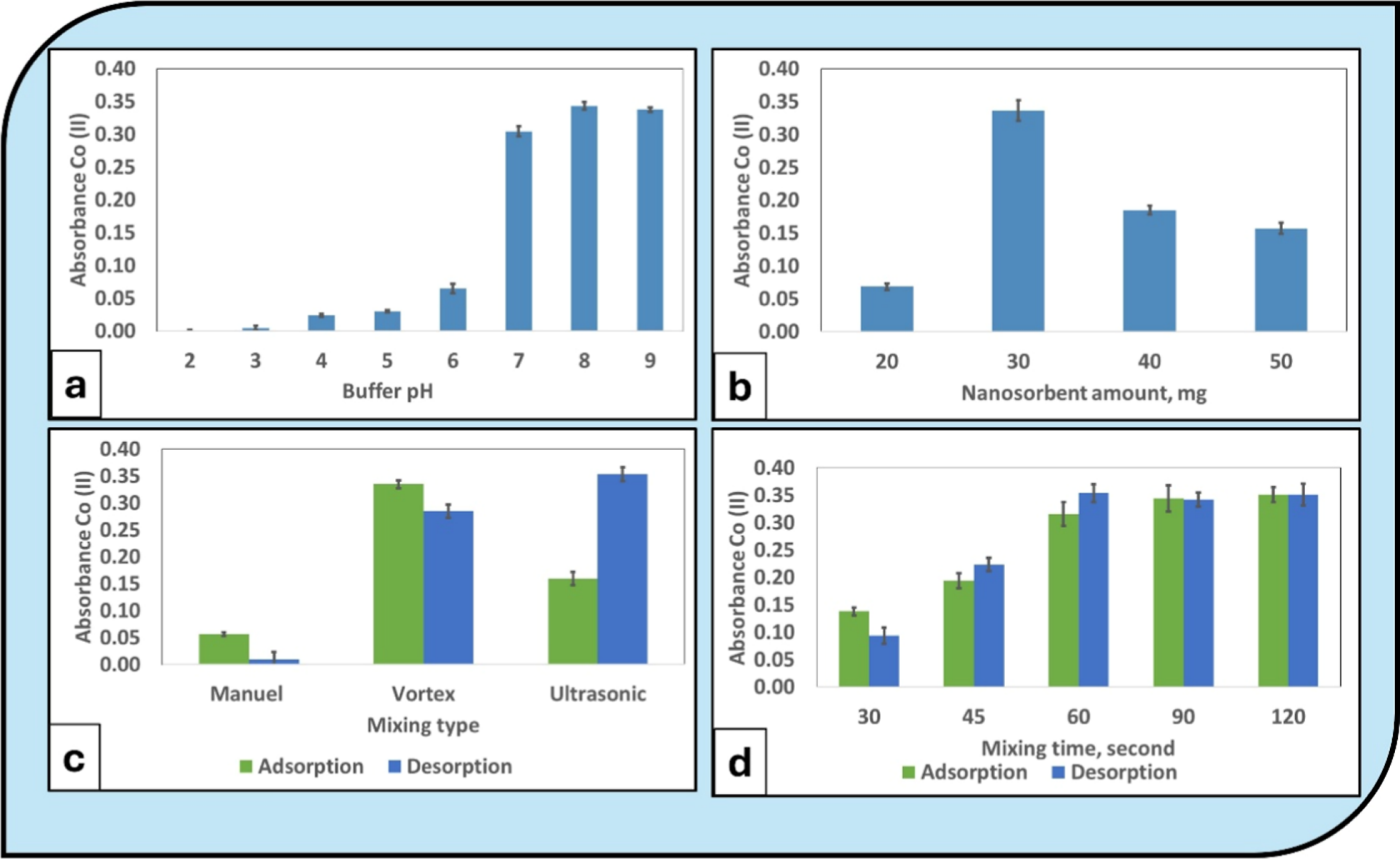
Effect of experimental parameters on Co­(II) extraction performance using Fe_3_O_4_-ALP: (a) influence of solution pH, (b) sorbent amount, (c) mixing type, and (d) mixing time. All optimization experiments were performed using a Co­(II) concentration of 250 ng mL^–1^ (500 ng mL^–1^ for the pH optimization series) and a sample volume of 40 mL (*n* = 3). Error bars represent ±1 standard deviation.

The strong suppression of extraction at low pH can be attributed to competitive protonation of surface-active sites, which limits effective interaction between Co­(II) ions and the sorbent surface. As pH increases, the gradual deprotonation of surface hydroxyl groups enhances electrostatic attraction and promotes surface complexation, leading to improved adsorption efficiency.^18^

At pH values above neutrality, although signal intensity remained high, excessively alkaline conditions were avoided to minimize potential hydrolysis of Co­(II) species and ensure analytical stability. Therefore, pH 8 was selected as the optimal working condition for subsequent experiments.

#### 3.2.3 Optimization of Sorbent Amount

The effect of sorbent amount on Co­(II) extraction efficiency was evaluated in the range of 20–50 mg (Figure 5b). Signal intensity increased markedly when the sorbent amount was raised from 20 to 30 mg, indicating that an insufficient number of active sites limited adsorption at lower dosages.

A maximum analytical response was observed at 30 mg, suggesting that this amount provided an adequate density of surface-active sites for efficient Co­(II) uptake under the selected sample volume. Increasing the sorbent amount beyond 30 mg resulted in a gradual decline in signal intensity.

This decrease at higher dosages may be attributed to particle aggregation and reduced effective dispersion in the sample matrix, which can limit accessible surface area and hinder mass transfer. Additionally, excessive sorbent quantities may dilute the desorbed analyte into a relatively larger solid phase, leading to reduced postextraction signal intensity.

Based on these observations, 30 mg was selected as the optimal sorbent amount for subsequent experiments.

#### 3.2.4 Optimization of Mixing Type and Time

The influence of mixing conditions on Co­(II) extraction efficiency was evaluated to ensure effective dispersion of the Fe_3_O_4_-ALP sorbent and efficient analyte transfer during both adsorption and desorption stages. All experiments were performed under previously optimized pH (8.0) and sorbent amount (30 mg) conditions.

Three agitation modes—manual shaking, vortex mixing, and ultrasonic treatment—were comparatively investigated (Figure 5c). The results clearly indicate that the optimal mixing strategy differs for adsorption and desorption steps. For the adsorption stage, vortex mixing provided the highest analytical signal. This suggests that vortex agitation enables efficient dispersion of the perlite sorbent within the aqueous phase, thereby maximizing surface contact and accelerating mass transfer between Co­(II) ions and active adsorption sites. In contrast, during the desorption step, ultrasonic treatment yielded the highest signal intensity. The enhanced desorption efficiency under ultrasonic conditions is attributed to the cavitation effect, which promotes disruption of analyte–sorbent interactions and facilitates rapid release of Co­(II) ions into the eluent phase. Manual shaking resulted in significantly lower signals for both stages, indicating insufficient mass transfer under mild agitation conditions. Accordingly, vortex mixing was selected as the optimal agitation mode for adsorption, while ultrasonic mixing was chosen for desorption.

The influence of mixing time was evaluated in the range of 30–120 s (Figure 5d). For adsorption, the analytical signal increased progressively up to 60 s, after which a plateau behavior was observed, indicating attainment of adsorption equilibrium. Therefore, 60 s was selected as the optimal adsorption time. For desorption, signal intensity continued to increase up to 90 s, reflecting gradual disruption of analyte–sorbent interactions. Beyond 90 s, no significant improvement was observed, indicating completion of the desorption process. Thus, 90 s was selected as the optimal desorption time. These findings demonstrate that adsorption and desorption stages require different mixing dynamics to achieve maximum analytical performance within the DSPE-FAAS procedure.

#### 3.2.5 Optimization of Eluent Type and Volume

The desorption step is critical in DSPE-FAAS because the eluent must efficiently disrupt Co­(II)-sorbent interactions and quantitatively transfer the retained analyte into a small final volume suitable for instrumental measurement. Therefore, both eluent type and eluent volume were optimized to maximize analytical signal while maintaining operational simplicity and compatibility with FAAS.

Three different eluents—HNO_3_, HCl, and CH_3_COOH—were evaluated under identical conditions. Among the tested eluents, HNO_3_ provided the highest analytical signal, whereas CH_3_COOH produced the lowest response, indicating insufficient desorption strength under the applied conditions. Compared with HCl, HNO_3_ yielded a 1.6-fold higher signal, demonstrating superior desorption efficiency for Co­(II) from the Fe_3_O_4_-ALP surface. The enhanced performance of HNO_3_ can be attributed to its stronger ability to protonate surface-active sites and destabilize surface complexes, thereby facilitating rapid and efficient release of Co­(II) into the eluent phase. Based on these results, HNO_3_ was selected as the optimal eluent for subsequent experiments.

Following the selection of HNO_3_, the eluent volume was optimized to achieve high desorption efficiency without compromising enrichment. The analytical signal increased as the eluent volume was reduced, reaching a maximum at 0.3 mL, which reflects the most favorable balance between quantitative desorption and minimal dilution of the analyte. Volumes higher than 0.3 mL resulted in lower signal intensity due to dilution of the desorbed Co­(II), despite effective elution. Therefore, 0.3 mL of 5 mol L^–1^ HNO_3_ was chosen as the optimum eluent volume. Because the optimized desorption was performed using a small volume (0.3 mL) of 5 mol L^–1^ HNO_3_, the eluate was subsequently diluted before FAAS measurement in order to prevent potential damage to the instrumental components and ensure stable nebulization conditions. This dilution step was performed solely to protect the FAAS system and did not affect the relative comparison or optimization results.

### 3.3 Analytical Figures of Merit

The analytical performance of the proposed DSPE-FAAS method was systematically assessed under optimized conditions in terms of linear dynamic range, limit of detection (LOD), limit of quantification (LOQ), LOD improvement factor (defined as the ratio of the direct FAAS LOD to the DSPE-FAAS LOD; abbreviated LIF), calibration linearity, and precision. A strong linear response was obtained over the concentration range of 5–250 ng mL^–1^ for Co­(II), with a correlation coefficient higher than 0.999, confirming excellent proportionality between absorbance and analyte concentration following the preconcentration step. This working range is well-suited for trace-level cobalt monitoring in environmentally relevant aqueous systems, including groundwater and rock leachate samples. The LOD and LOQ values, calculated according to the 3σ/m and 10σ/m criteria, were determined to be 0.626 ng mL^–1^ and 2.086 ng mL^–1^, respectively. These low values demonstrate the high sensitivity of the developed procedure and reflect the effective preconcentration capability of the Fe_3_O_4_-ALP sorbent.

A LOD improvement factor (LIF) of 122 was achieved, calculated as the ratio of the direct FAAS LOD (76.9 ng mL^–1^) to the DSPE–FAAS LOD (0.626 ng mL^–1^), reflecting the effective preconcentration capability of Fe_3_O_4_-ALP under dispersive extraction conditions. This substantial signal amplification significantly extends the applicability of FAAS, a technique typically limited by moderate intrinsic sensitivity compared to ICP-based methods. The precision of the method, expressed as relative standard deviation (RSD), ranged from 4.4% to 8.1%, indicating satisfactory repeatability at trace concentration levels. The stable analytical response confirms the robustness of the adsorption–desorption process within the optimized DSPE protocol.

Overall, these analytical figures of merit demonstrate that the proposed DSPE-FAAS method provides a sensitive, reliable, and cost-effective strategy for trace Co­(II) determination in geochemically complex aqueous matrices.

The analytical performance of the developed DSPE-FAAS method was benchmarked against previously reported preconcentration methods for Co­(II) determination, as summarized in Table 2. The proposed Fe_3_O_4_-ALP-based method achieved a LOD of 0.626 ng mL^–1^, which is competitive with the most sensitive methods in the table, including ox-MWCNTs/BNBATT (0.60 ng mL^–1^), PAMAM-Fe_3_O_4_ (0.8 ng mL^–1^), and Fe_3_O_4_-AEAPTMS (0.5 ng mL^–1^), and substantially lower than magnetic *Pinus pinea* (19.3 ng mL^–1^) and PVC-TADAP (1.3 ng mL^–1^). Notably, the LIF of 122 is among the highest values reported for DSPE-FAAS-based Co­(II) determination, markedly exceeding the *P. pinea* bioadsorbent (PF = 25) and PVC-TADAP (PF = 40), and comparable to the PAMAM-Fe_3_O_4_ system (EF = 110). The linear range of 5–250 ng mL^–1^ is well-suited to environmentally relevant Co­(II) concentrations in natural waters. A key distinguishing feature of the present method is its validation in groundwater and rock leachate matrices, which represent geochemically complex and analytically demanding sample types not previously reported for DSPE-FAAS-based Co­(II) methods. Furthermore, the use of volcanic perlite—a geologically derived, low-cost mineral sorbent—requiring no chelating agents or multistep organic functionalization offers a practical sustainability advantage over synthetic sorbents such as PAMAM dendrimers or carbon nanotube-based materials commonly reported in the literature.

**Table 2:** Comparison of Selected Preconcentration Methods for Co­(II) Determination by FAAS and Related Techniques

sorbent/method for Co(II)	detection	LOD (ng mL^–1^)	linear range (ng mL^–1^)	LIF/EF/PF^a^	real samples	refs
Fe_3_O_4_-ALP (DSPE)	FAAS	0.626	5–250	LIF = 122 EF = 67 PF = 133	groundwater, Rock leachate	this study
ox-MWCNTs/BNBATT (VA-DμSPE)	FAAS	0.60	2–500	PF = 200	water, juice, food	^19^
magnetic Pinus pinea (DSPE)	FAAS	19.3	2–500	PF = 25	spring, mineral, process water	^20^
Fe_3_O_4_@coPPy-PTH (MSPE)	MIS-FAAS	0.17	0–10	ER^b^= 88.2%	beer, wine, mineral water	^21^
sol–gel thiocyanatopropyl silica	Online FI-FAAS	0.5	1.7–80	EF = 105	river, lake water	^22^
activated carbon (grape stalk)	HR-CS FAAS	0.27	200–4000	PF = 150	water, food	^23^
PVC-TADAP (Online SPE)	FAAS	1.3	2–150	PF = 40	tap water, wastewater, food	^24^
Fe_3_O_4_-AEAPTMS (SPE)	FAAS	0.5	1–200 μg L^–1^		food, water	^25^
PAMAM-Fe_3_O_4_ (DSPE)	FAAS	0.8	2.5–250	EF = 110	tap water, tea	^26^
ox-MWCNTs (UA-DMSPE)	FAAS	0.30	1–200	PF = 200	environmental waters	^27^
Fe-MNP-PAMAM (SPE)	FAAS	1.1		LIF = 101×	tea	^28^

a PF: preconcentration factor; EF: enrichment factor; LIF: LOD improvement factor, i.e., direct FAAS LOD/DSPE–FAAS LOD.

b ER: extraction recovery. Fe_3_O_4_-AEAPTMS = [3-(2-aminoethyl amino)­propyl]­trimethoxysilane-functionalized Fe_3_O_4_ (ref 25); PAMAM-Fe_3_O_4_ = phosphonate ester-functionalized PAMAM dendrimer-coated magnetite (ref 26); ox-MWCNTs = oxidized multiwalled carbon nanotubes (ref 27); Fe-MNP–PAMAM = PAMAM–OH-encapsulated iron magnetic nanoparticles (ref 28). LOD for ref. Twenty-five reported in μg L^–1^.

### 3.4 Real Samples Application

The practical applicability of the developed DSPE-FAAS method was evaluated using groundwater and rock leachate samples to assess its performance under geochemically relevant conditions. Unlike synthetic solutions, natural waters represent complex hydrochemical systems where trace cobalt concentrations are governed by mineralogical controls, redox conditions, and water–rock interaction processes. Therefore, validation in such matrices is essential to demonstrate analytical robustness.

Groundwater samples collected from three different locations were analyzed after appropriate filtration and dilution. Recovery experiments were performed using matrix-matched calibration standards prepared in the respective sample matrices to compensate for potential matrix effects. Spiked samples at three concentration levels (10, 50, and 100 ng mL^–1^) were performed to evaluate matrix effects arising from dissolved salts, competing cations, and natural organic matter. The obtained recovery values ranged between 84.4% and 110.4% (Table 3), indicating satisfactory accuracy across distinct hydrochemical environments. The acceptable standard deviation values confirm that the adsorption–desorption equilibrium of Co­(II) on Fe_3_O_4_-ALP remains stable even in matrices characterized by variable ionic strength and potential competitive sorption processes.

**Table 3:** Recovery of Co­(II) from Groundwater and Rock Leachate Samples

sample matrix	Co(II) spiked conc. (ng mL^‑1^)	recovery (%)	± SD^a^
groundwater-Tusba	10	95.8	±5.4
	50	101.2	±5.8
	100	98.7	±4.4
groundwater-Edremit	10	96.5	±6.1
	50	101.3	±3.2
	100	104.4	±2.2
groundwater-Ipekyolu	10	104.6	±6.1
	50	84.4	±5.2
	100	92.5	±4.8
rock leachate	10	96.6	±3.8
	50	101.1	±5.3
	100	105.2	±6.0

a Standard deviation of three replicate measurements (*n* = 3).

Rock leachate samples were prepared to simulate geogenic cobalt mobilization during water–rock interaction. In natural geological systems, cobalt is commonly associated with mafic and ultramafic lithologies and may occur in sulfide phases or adsorbed onto Fe/Mn (oxyhydr)­oxides.^29^ During weathering and leaching processes, partial dissolution of these mineral phases can release Co into aqueous environments. The laboratory leaching procedure, therefore, provides a controlled analogue of natural geochemical mobilization pathways.

For rock leachate matrices, recoveries were found between 96.6% and 105.2%, demonstrating that the magnetic Fe_3_O_4_-ALP sorbent efficiently captures dissolved cobalt even in solutions derived from mineral dissolution. The strong performance in these samples is particularly noteworthy, as such matrices may contain elevated concentrations of iron, manganese, calcium, magnesium, and silicate species that could potentially interfere with adsorption-based extraction systems.

The consistent analytical response observed in both groundwater and rock-derived leachates indicates that the proposed method tolerates geochemically complex matrices without significant signal suppression or enhancement artifacts. From a geochemical standpoint, this reliability is critical for investigating cobalt transport in mineralized terrains, weathering profiles, and mine-impacted environments, where trace-level determination must be achieved despite variable physicochemical conditions.

Overall, the real sample results confirm that the Fe_3_O_4_-ALP-based DSPE-FAAS method constitutes a robust and geologically meaningful analytical tool for trace Co­(II) monitoring in natural aqueous systems influenced by mineralogical and hydrogeochemical controls.

## 4 Conclusion

In this study, acid-treated lamellar nanoperlite (ALP) and its Fe_3_O_4_-decorated analogue (Fe_3_O_4_-ALP) were successfully synthesized and applied as sorbents in a DSPE-FAAS method for trace Co­(II) determination. The lamellar aluminosilicate framework obtained through acid activation and calcination provided an efficient sorbent architecture with accessible surface sites, while Fe_3_O_4_ decoration further enhanced Co­(II) uptake and enabled convenient magnetic phase separation within the DSPE workflow.

Comprehensive characterization by SEM–EDX, FTIR, XRD, and BET analyses confirmed the successful magnetic functionalization and the formation of a mesoporous composite architecture with enhanced surface heterogeneity. Comparative experiments demonstrated that Fe_3_O_4_-ALP provided substantially higher signal enhancement than pristine ALP, confirming the beneficial role of magnetic decoration in improving adsorption efficiency.

Under optimized conditions, the developed DSPE-FAAS method exhibited excellent linearity (5–250 ng mL^–1^, *R* ^2^ > 0.999), low detection limits (LOD: 0.626 ng mL^–1^; LOQ: 2.086 ng mL^–1^), and a LOD improvement factor (LIF) of 122. The method showed satisfactory precision (RSD 4.4–8.1%) and demonstrated reliable performance in groundwater and rock leachate samples, where recovery values confirmed its tolerance toward geochemically complex matrices.

From an environmental analytical perspective, the use of a mineral-based, cost-effective sorbent in a DSPE-FAAS workflow offers a practical tool for trace Co­(II) monitoring in systems influenced by rock–water interaction and mineral weathering. The robustness of the method in matrices containing competing ions highlights its suitability for environmental monitoring and hydrogeochemical studies.

Overall, Fe_3_O_4_-ALP represents a cost-effective, environmentally compatible, and analytically capable sorbent for trace cobalt determination in environmental and geological applications.

## Supplementary Material



## Data Availability

All data generated or analyzed during this study are included in this published article. Additional data supporting the findings of this study are available from the corresponding author upon reasonable request.

## Notes

Tables S1–S9 present the effects of sorbent type, solution pH, sorbent amount, mixing type and time, and eluent volume on Co­(II) extraction efficiency. Calibration data and analytical performance parameters under optimized DSPE-FAAS conditions, as well as recovery results for groundwater and rock leachate samples, are also provided (PDF)
