# Supplementary material for: Synthesis and Application of Fe3O4–Modified Nano-Perlite as a Dispersive Sorbent for Co(II) Ion Preconcentration
Source: ACS Omega. 2026 May 14;11(20):30062–72. doi: 10.1021/acsomega.6c01728 (PMC13216951; doi:10.1021/acsomega.6c01728)
Supplement: Supplementary file 1 [file ao6c01728_si_001.pdf]

# Supporting Information

*Synthesis and Application of $Fe_3O_4$-Modified Nano-Perlite as a Dispersive Sorbent for Co(II) Ion Preconcentration*

Ali Kılıçer

*Department of Geological Engineering, Van Yüzüncü Yıl University, Van, Türkiye*

Corresponding author: alikilicer@yyu.edu.tr

This Supporting Information provides analytical data for the optimization of the DSPE–FAAS procedure for Co(II) determination using $Fe_3O_4$–ALP. Unless otherwise stated, all optimization experiments were performed with a Co(II) concentration of 250 ng mL⁻¹ and a sample volume of 40 mL (n = 3). The pH and effect of sorbent optimization were conducted at 500 ng mL⁻¹ Co(II). The optimum condition is indicated by an asterisk (*). Mean absorbance values are reported to 4 decimal places.

**Table S1. Effect of sorbent type on Co(II) extraction efficiency.**

| Sorbent | Signal Enhancement Factor[a] |
|---|---|
| ALP | 26 |
| $Fe_3O_4$–ALP | 45 |

[a]*Signal enhancement factor = signal after DSPE / direct FAAS signal at the same Co(II) concentration. Conditions: 500 ng mL⁻¹ Co(II), 40 mL sample, 30 mg sorbent, natural pH, n = 3.*

**Table S2. Effect of solution pH on Co(II) extraction efficiency using $Fe_3O_4$–ALP.**

| pH | Mean Absorbance | %RSD | Enhancement Factor |
|---|---|---|---|
| 2 | 0.0013 | 2.7 | 0.10 |
| 3 | 0.0031 | 8.1 | 0.23 |
| 4 | 0.0238 | 7.4 | 1.78 |
| 5 | 0.0300 | 4.7 | 2.26 |
| 6 | 0.0640 | 8.8 | 4.80 |
| 7 | 0.3045 | 2.6 | 22.8 |
| 8* | 0.3433 | 1.6 | 25.7 |
| 9 | 0.3375 | 1.0 | 25.3 |

*Signal enhancement factor = signal after DSPE / direct FAAS signal at the same Co(II) concentration. Conditions: 500 ng mL⁻¹ Co(II), 40 mL sample, 30 mg $Fe_3O_4$–ALP, n = 3. The elevated %RSD at pH 3 reflects near-baseline absorbance at strongly acidic conditions. *Optimum pH.*

**Table S3. Effect of $Fe_3O_4$–ALP sorbent amount on Co(II) extraction efficiency.**

| Sorbent (mg) | Mean Absorbance | %RSD | Enhancement Factor |
|---|---|---|---|
| 20 | 0.0686 | 7.7 | 6.32 |
| 30* | 0.3367 | 4.5 | 31.04 |
| 40 | 0.1828 | 3.2 | 16.86 |
| 50 | 0.1548 | 4.6 | 14.27 |

*Signal enhancement factor = signal after DSPE / direct FAAS signal at the same Co(II) concentration. Conditions: 250 ng mL⁻¹ Co(II), 40 mL, pH 8, n = 3. *Optimum condition.*

**Table S4. Effect of mixing type on Co(II) adsorption and desorption efficiency.**

| Mixing Type | Mean Absorbance (Adsorption) | Mean Absorbance (Desorption) | Enhancement Factor | %RSD (Adsorption) |
|---|---|---|---|---|
| Manual | 0.0563 | 0.0100 | 5.16 | 5.7 |

| | | | | |
|---|---|---|---|---|
| Vortex* (adsorption) | 0.3350 | 0.208 | 30.71 | 2.1 |
| Ultrasonic* (desorption) | 0.1596 | 0.3536 | 14.63 | 7.8 |

*Signal enhancement factor = signal after DSPE / direct FAAS signal at the same Co(II) concentration. Conditions: 250 ng mL $^{-1}$ Co(II), 40 mL, pH 8, 30 mg $Fe_3O_4$–ALP, n = 3. *Vortex: optimum for adsorption; Ultrasonic: optimum for desorption.*

**Table S5. Effect of mixing time on Co(II) adsorption and desorption efficiency.**

| Time (s) | Mean Absorbance (Adsorption) | Mean Absorbance (Desorption) | Enhancement Factor | %RSD (Adsorption) |
|---|---|---|---|---|
| 30 | 0.1379 | 0.0935 | 13.13 | 5.4 |
| 45 | 0.1939 | 0.2235 | 18.47 | 7.1 |
| 60* (adsorption) | 0.3159 | 0.3540 | 30.09 | 6.9 |
| 90* (desorption) | 0.3442 | 0.3422 | 32.78 | 6.8 |
| 120 | 0.3412 | 0.3413 | 32.50 | 7.5 |

*Signal enhancement factor = signal after DSPE / direct FAAS signal at the same Co(II) concentration. Conditions: 250 ng mL $^{-1}$ Co(II), 40 mL, pH 8, 30 mg $Fe_3O_4$–ALP, Vortex (adsorption), Ultrasonic (desorption), n = 3. *Optimum: 60 s (adsorption), 90 s (desorption).*

**Table S6. Effect of $HNO_3$ eluent volume on Co(II) desorption efficiency.**

| $HNO_3$ Volume (mL) | Mean Absorbance | %RSD | Enhancement Factor |
|---|---|---|---|
| 0.3* | 0.3792 | 2.5 | 34.79 |
| 0.4 | 0.2606 | 4.2 | 23.91 |
| 0.5 | 0.2215 | 7.8 | 20.32 |
| 0.75 | 0.1619 | 2.1 | 14.85 |
| 1.0 | 0.1198 | 4.5 | 10.99 |

*Signal enhancement factor = signal after DSPE / direct FAAS signal at the same Co(II) concentration. Conditions: 250 ng mL $^{-1}$ Co(II), 40 mL, pH 8, 30 mg $Fe_3O_4$–ALP, Vortex 60 s (adsorption), Ultrasonic 90 s (desorption), 5 mol L $^{-1}$ $HNO_3$ as eluent, n = 3. *Optimum condition.*

**Table S7. Calibration data for Co(II) determination by DSPE–FAAS under fully optimized conditions.**

| Co(II) (ng mL $^{-1}$) | Mean Absorbance | %RSD |
|---|---|---|
| 0 (blank) | 0.0011 | 0.5 |
| 5 | 0.0070 | 8.1 |
| 10 | 0.0155 | 5.1 |
| 50 | 0.0718 | 6.2 |
| 100 | 0.1450 | 4.4 |
| 250 | 0.3890 | 6.4 |

*Calibration data measured after DSPE preconcentration under fully optimized conditions. Linear range: 5–250 ng mL $^{-1}$; $R^2 > 0.999$. LOD = 0.626 ng mL $^{-1}$ (3σ/m); LOQ = 2.086 ng mL $^{-1}$ (10σ/m); LOD improvement factor (LIF) = 122 (direct FAAS LOD / DSPE–FAAS LOD = 76.9 / 0.626).*

**Table S8. Summary of optimized DSPE–FAAS conditions and analytical performance parameters.**

| Parameter | Value |
|---|---|
| Linear range (ng mL $^{-1}$) | 5–250 |
| Correlation coefficient ($R^2$) | >0.999 |
| LOD (ng mL $^{-1}$) | 0.626 |
| LOQ (ng mL $^{-1}$) | 2.086 |

| | |
|---|---|
| LOD improvement factor (LIF) | 122 (= 76.9 / 0.626) |
| Preconcentration factor (PF) | 133 (= 40 mL / 0.3 mL) |
| Enhancement factor (EF) | 67 (= DSPE slope / direct FAAS slope) |
| Precision, %RSD (n = 3) | 4.4–8.1 |
| Direct FAAS LOD (ng $mL^{-1}$) | 76.9 |
| Co(II) conc. in optimization (ng $mL^{-1}$) | 250 (pH optimization: 500) |
| Sample volume (mL) | 40 |
| Sorbent amount (mg) | 30 |
| Optimum pH | 8 |
| Adsorption: mixing type/time | Vortex / 60 s |
| Desorption: mixing type/time | Ultrasonic / 90s |
| Eluent type and volume | 0.3 mL $HNO_3$ |

*LOD and LOQ by 3σ/m and 10σ/m criteria. Three complementary metrics were used to characterize the preconcentration performance of the method. The preconcentration factor (PF), defined as the ratio of the sample volume to the eluent volume, was calculated as 133 (40 mL / 0.3 mL). The enhancement factor (EF), defined as the ratio of the DSPE–FAAS calibration slope to the direct FAAS calibration slope expressed in equivalent concentration units, was determined as 67. The LOD improvement factor (LIF), defined as the ratio of the direct FAAS LOD to the DSPE–FAAS LOD, was calculated as 122 (76.9 / 0.626 ng $mL^{-1}$).*

**Table S9. Recovery of Co(II) from groundwater and rock leachate samples by DSPE–FAAS (n = 3).**

| Sample Matrix | Co(II) Added (ng $mL^{-1}$) | Recovery (%) | SD (±) | n |
|---|---|---|---|---|
| Groundwater – Tusba | 10 | 95.8 | 5.4 | 3 |
| | 50 | 101.2 | 5.8 | 3 |
| | 100 | 98.7 | 4.4 | 3 |
| Groundwater – Edremit | 10 | 96.5 | 6.1 | 3 |
| | 50 | 101.3 | 3.2 | 3 |
| | 100 | 104.4 | 2.2 | 3 |
| Groundwater – Ipekyolu | 10 | 104.6 | 6.1 | 3 |
| | 50 | 84.4 | 5.2 | 3 |
| | 100 | 92.5 | 4.8 | 3 |
| Rock leachate | 10 | 96.6 | 3.8 | 3 |
| | 50 | 101.1 | 5.3 | 3 |
| | 100 | 105.2 | 6.0 | 3 |

*Conditions: pH 8, 30 mg $Fe_3O_4$–ALP, 40 mL, Vortex 60 s (adsorption), Ultrasonic 90 s (desorption), 0.3 mL of 5 mol $L^{-1}$ $HNO_3$. SD = standard deviation (n = 3). Recovery values were determined using matrix-matched calibration standards prepared in the respective sample matrices.*
